# Circulating antibody signatures against *Mycobacterium tuberculosis* antigens are associated with severe and mild tuberculosis

**DOI:** 10.3389/fimmu.2026.1897502

**Published:** 2026-08-25

**Authors:** Agustín D. Vitti, Rocío Zuazo, Ana J. Bazán Bouyrie, Sergio I. Nemirovsky, Candela Martin, Camila B. Martinena, Nancy L. Tateosian, Lorena M. Ciallella, Graciela C. de Casado, Domingo J. Palmero, Juan L. Iovanna, Verónica E. García, Nicolás O. Amiano

**Affiliations:** 1 Laboratory of Immunity and Tuberculosis, Institute of Biological Chemistry of the Faculty of Exact and Natural Sciences, University of Buenos Aires (UBA) - National Council of Scientific and Technical Investigations (CONICET), Buenos Aires, Argentina; 2 Department of Biological Chemistry, Faculty of Exact and Natural Sciences, Universidad de Buenos Aires (UBA), Buenos Aires, Argentina; 3 Division of Tisioneumonology, Hospital F.J. Muñiz, Buenos Aires, Argentina; 4 Cancer Research Center of Marseille, National Institute of Health and Medical Research (INSERM) - National Centre for Scientific Research (CNRS) - Institute Paoli-Calmettes - Aix-Marseille University, Marseille, France; 5 Department of Organic Chemistry, Faculty of Exact and Natural Sciences (FCEN). University of Buenos Aires (UBA), Buenos Aires, Argentina

**Keywords:** antibody profiling, antigen recognition, clinical phenotyping, disease severity, DosR regulon antigens, immune correlates, serological biomarkers, tuberculosis

## Abstract

**Introduction:**

Tuberculosis (TB), caused by *Mycobacterium tuberculosis*, is a highly heterogeneous disease driven by the pathogen’s adaptive mechanisms to subvert the host immune response. Identifying biomarkers that can stratify disease severity remains a major challenge.

**Methods:**

In this work, we analyzed a large cohort of patients with active TB by integrating clinical, radiological, and laboratory variables. Using Factor Analysis of Mixed Data (FAMD) to define a range of severity-associated phenotypes, two principal components of variation, related to systemic and pulmonary severity were identified and combined in an overall score. Plasma circulating IgG responses against ESAT-6/CFP-10, Ag85A, HspX and Rv2626c -four key antigens with distinct behaviors during metabolic switches of *Mtb*- were evaluated as antibody signatures.

**Results:**

Individual antibody responses showed limited associations with severity. However, specific antibody signatures presented a significantly higher effect. A signature defined by IgG responses against ESAT-6/CFP-10, Ag85A, and HspX (excluding Rv2626c) was associated with higher severity scores and features of advanced pulmonary disease. In contrast, signatures containing antibodies against Rv2626c were mainly associated with milder phenotypes.

**Conclusion:**

These findings demonstrate that antigen-specific IgG signatures show significant associations with specific forms of TB pathology. Additional prospective and longitudinal studies will be required to study their potential as biomarkers.

## 1 Introduction

*Mycobacterium tuberculosis* (*Mtb*) remains a major global health challenge, causing more than one million deaths each year and representing the leading cause of death from a single infectious agent (1). This pathogen is able to evade the host immune response, leading to either tuberculosis disease (TB) or latent tuberculosis infection (LTBI) (2).

In its active state, *Mtb* displays an aerobic metabolism. However, during infection, bacilli are phagocytosed by macrophages, which are subsequently activated by T cells (3) and aggregate to form granulomas (4). At this point *Mtb* senses the resulting respiratory stress, slowing down its replication and altering its patterns of protein expression and secretion to enter a state of non-replicative persistence (NRP) (5). During early NRP, *Mtb* induces its Dormancy survival regulon (DosR), highly upregulating proteins which counter the host immune response (6, 7) and preparing for a later, metabolically inactive NRP, with the potential for future reactivation (8, 9).

Traditionally, NRP has been associated with LTBI (5). However, advanced imaging techniques have identified large hypoxic areas in the lungs of patients with active TB (10). Moreover, TB development is complex, usually starting as a transient “primary TB” that affects the base of the lungs with the typical granulomatous inflammation known to induce NRP (4). Before resolution, bacilli may disseminate via lymphohematogenous pathways to extrapulmonary sites and later reseed the lungs, leading to post-primary TB (11). Alternatively, reinfection may also occur, resulting in a similar outcome (4). This last form of TB usually begins as an asymptomatic alveolitis at the lung apices, leading to caseous pneumonia and the formation of aerated cavities from which *Mtb* is released in large numbers, promoting transmission to new hosts (12). In some cases, primary TB may lead to infection of lymph nodes (13) and pleura (14), two highly inflammatory environments in which most bacilli are likely to be in a NRP state. Moreover, *Mtb* can occasionally compromise other organs; the most severe manifestation is miliary TB, usually caused by rupture of an infection focus leading to widespread hematogenous dissemination and the formation of small nodules throughout the body (15).

During infection, adaptive immunity can provide information about the state of bacilli by targeting differentially expressed antigens (16). However, antigen expression is not sufficient for development of immunity. Antigens must be available for lymphocyte recognition and virulence factors may hamper the response (17). In particular, *Mtb* has been shown to alter B cells, leading to impaired proliferation and immunoglobulin- and cytokine-production (18). Nevertheless, severe TB has been associated with increased levels of B-cell–activating cytokines (19) and specific antibodies against *Mtb* can be detected in many TB patients (20–27).

Several antigens have been reported to contribute to the adaptability of *Mtb*. Among these, ESAT-6, CFP-10, Ag85A, HspX, and Rv2626c are key players in human TB (28). ESAT-6 and CFP-10 (E6/C10) are co-expressed proteins from *Mtb* that play a crucial role in its virulence, being induced during aerobic growth but continuously secreted by metabolically active bacilli (29, 30). ESAT-6 is a virulence factor with multiple immune evasion functions (31). CFP10 enables ESAT-6 secretion (32) and can also recruit neutrophils (33), which promote disease progression (34). Ag85A plays a role in synthesizing *Mtb* hydrophobic cell wall and in binding to fibronectin (35), and is required for intracellular growth (36). This antigen is expressed and secreted by replicating bacilli but is downregulated (29) and retained during NRP state, playing a significant role in the formation of lipid bodies (37). Moreover, Ag85A can be induced once more by reactivation (9). HspX is a chaperone that is induced during early NRP state by the DosR transcription factor (3, 38), slowing down the replication of *Mtb* (39). However, HspX is also secreted during disease (40) and plays a role in the induction of tolerogenic myeloid cells from bone marrow progenitors (41). Finally, Rv2626c is a secreted protein also induced by DosR during NRP state (3, 38). This protein enhances E6/C10 secretion (42) and modulates macrophage functions, recruiting monocytes to the lung, promoting their polarization toward tolerogenic phenotypes (7) and inducing necrosis (6).

In this study, we integrated clinical, radiological, and laboratory data using Factor Analysis of Mixed Data (FAMD) to define severity-associated phenotypes in a large cohort of patients with active TB. Furthermore, we characterized circulating IgG responses against ESAT-6/CFP-10, Ag85A, HspX, and Rv2626c as antibody signatures. Our findings demonstrate that specific combinations of antigen-specific IgG responses are associated with distinct clinical manifestations of TB. Future prospective and longitudinal studies are needed to determine their potential as biomarkers for disease stratification.

## 2 Materials and methods

### 2.1 Study populations

Plasma samples were collected and stored at -80 °C during a period ranging from 2010 to 2024. 505 Individuals with active tuberculosis (TB) were recruited at Hospital Dr. F. Muñiz (Buenos Aires, Argentina). 23 healthy controls (HC) were also enrolled.

Inclusion criteria were a) adult (over 18 years old) men and women with an active TB diagnosis based on clinical and radiological assessment and confirmed by positive culture and b) adult men and women with no known exposure to *Mtb*. Exclusion criteria were a) HIV positive infection or positive serology for other viral (e.g., hepatitis A, B or C), or bacterial infections (e.g., leprosy, syphilis, etc); b) patients with diabetes, cancer, autoimmune diseases or other conditions that may affect the immune system of the individual; c) pregnant women and d) children. Among the population of TB patients, we also excluded: a) patients with multidrug-resistant tuberculosis (MDR) infection or extremely drug resistant (XDR) tuberculosis and b) patients with more than seven consecutive days of anti-TB treatment (rifampicin, pyrazinamide, ethambutol and isoniazid). Among HC population, we also excluded individuals with: a) positive result for the QuantiFERON-TB Gold Plus assay (Qiagen, Germany, USA) b) history of exposure to *Mtb*. All enrolled participants had received BCG vaccination.

Information regarding demographic data was obtained at the time of recruitment through a structured questionnaire. Clinical, radiological, and laboratory data were provided by primary care workers. AFB smear results were semi-quantitatively graded as - (less than 9 bacilli in 100 fields), + (10–99 bacilli in 100 fields), ++ (50–500 bacilli in the first 50 fields), and +++ (more than 200 bacilli in the first 20 fields), according to standard smear microscopy grading criteria. Radiological lesions were classified as unilateral or bilateral and semi-quantitatively graded by the number of X-ray zones (upper, mid and lower) presenting lesions. For unilateral lesions: + (1 zone) and ++ (more than 1 zone) were used. For bilateral lesions: + (2 zones), ++ (3 or 4 zones) and +++ (5 or 6 zones) were used.

### 2.2 Antigen purification

Ag85A, HspX and Rv2626 were expressed in *Escherichia coli* BL21 (DE3) and a fusion protein of antigens ESAT-6 and CFP-10 (E6/C10) was expressed in *Escherichia coli* M15 (Qiagen, USA). Purification of proteins was performed using a nickel nitrilotriacetic system (Ni-NTA Agarose, Qiagen, USA), following the manufacturer s instructions. After purity control, antigens were concentrated using Amicon (Merck Millipore) commercial columns and Detoxi-Gel Endotoxin Removing Resin (Pierce, USA) was used to remove endotoxin traces.

### 2.3 Antibody - enzyme-linked immunosorbent assay

Serum levels of specific IgG levels against E6/C10, Rv2626c, HspX and Ag85A antigens were measured by ELISA in plasma obtained from heparinized whole blood. High binding ELISA plates (Greiner Bio-one. Cat. No.: 675061) were used for the assay, coating separated wells with each recombinant antigen at 2.5μg/mL in 0.05 M carbonate-bicarbonate buffer pH: 9.5 and leaving uncoated wells as control for unspecific binding. After overnight incubation at 4 °C, plates were blocked with phosphate-buffered saline containing 1% bovine serum albumin (BSA) for 1 h at room temperature. Samples were then added at a 1:100 dilution in blocking solution, with one well coated with each recombinant antigen and one uncoated well per sample, followed by incubation for 2 h at 37 °C. Then, a secondary antibody (biotin mouse anti-human IgG - BD Pharmingen, USA) was added, following 1h of incubation at room temperature. After this, high sensitivity streptavidin - horseradish peroxidase enzyme (Thermo Fisher Scientific, USA) was incorporated, with 30’ of incubation, followed by 3,3′5,5′-tetramethylbenzidine (TMB) substrate (Sigma-Aldrich, USA). Sulfuric acid 2N was used to stop the enzyme reaction at 30 min and plate absorbance at 450nm was measured with a iMark BIO-RAD microplate reader. Finally, background absorbance (uncoated wells) was subtracted from antigen-coated wells.

Following measurements, cut-off points were established by ROC analysis, taking HC as negative controls. Then, antibody signatures were defined as the combinations of antigens recognized by each individual.

### 2.4 QuantiFERON-TB Gold Plus assay

QFT (Qiagen, Germany, USA) test and data interpretation were performed according to the manufacturer s instructions. QFT results were recorded positive if a single antigen tube (either TB1 or TB2) was ≥ 0.35 IU/mL as compared to the negative control (“Nil” tube). Healthy individuals with no known exposure to *Mtb* and a negative result for QFT were considered free of LTBI.

### 2.5 Factor analysis for mixed data

Dimensionality reduction of the clinical variables, which were of numerical, ordinal and binomial nature, was performed using a FAMD approach as implemented in FactoMineR (43). To be included in the analysis, patient data could not have more than 6 missing records on the selected variables. The remaining missing data were imputed using missMDA with the regularised iterative FAMD algorithm (44). Rows in the data matrix with imputed data were assigned reduced weights (W) in the analysis defined as the inverse of the square root of the number of missing values (n_{missing}_) plus one.


$$\text{w}=\frac{1}{\left\{\sqrt{\left\{{\text{n}}_{\left\{\text{missing}\right\}}+1\right\}}\right\}}$$

### 2.6 Statistical analysis

For all data sets normality was assessed using formal tests and visual inspection of Q–Q plots. When normality assumptions were met, two-tailed parametric tests for unpaired samples were used (t-test with Welch correction for pairwise comparisons or Brown-Forsythe and Welch ANOVA tests followed by Dunnett’s T3 multiple comparisons post-test, for multiple comparisons). For variables showing log-normal distribution, log transformation was applied prior to analysis. For sufficiently large sample sizes, normality was assumed based on the Central Limit Theorem. For small samples deviating from both normality and lognormality non-parametric tests were used (Mann-Whitney test, for pairwise comparisons or Kruskal – Wallis followed by Dunn’s multiple comparisons, for multiple comparisons). For categorical variables, Fisher’s exact test was used for 2×2 contingency tables; otherwise, the chi-square test was applied. Benjamini-Hochberg correction for multiple comparisons was applied to avoid potential inflation of the Type I error rate as indicated in figure legends. *P*-value < 0.05 as statistically significant. All analyses were performed using GraphPad Prism 10.2 software.

## 3 Results

### 3.1 Study participants

This study involved a total of 505 patients with active TB and 23 healthy controls without LTBI and without known exposure to *Mtb*. Demographic characteristics of enrolled TB patients are summarized in **Table 1**. All individuals had received BCG vaccination at birth.

**Table 1:** Population characteristics.

Study subjects characteristics		TB patients (TB) (N = 505)
Age (years), median (range)		31.7 (18–83)
Sex	Male	344 (68.1%)
	Female	161 (31.9%)
Country of origin	Argentina	278 (61.6%)
	South America (other)	173 (38.4%)

Values were expressed as number (percentage) for sex and country of origin.

### 3.2 Multivariate analysis to evaluate tuberculosis severity

**Figure 1:**
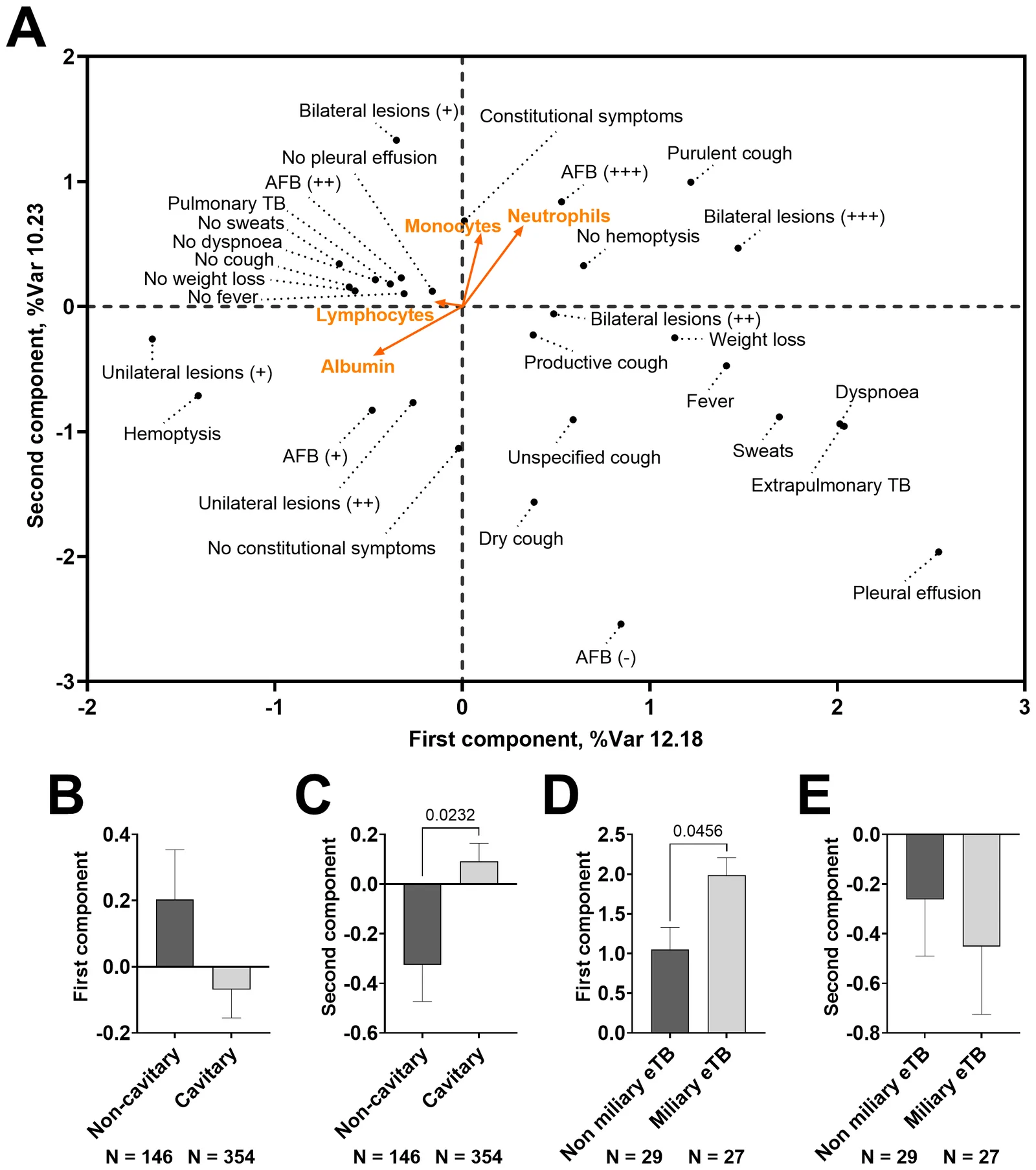
FAMD analysis of clinical, radiological, and laboratory severity variables. **(A)** Contribution of qualitative (black dots) and quantitative (orange arrows) variables to FAMD. First and second components are represented as coordinates, indicating the percentage of variance explained by each (12.18% and 10.23%). AFB smear results were semi-quantitatively graded as - (negative), + (low), ++ (moderate), and +++ (high bacillary load) according to standard smear microscopy grading criteria. Radiological lesions were classified as unilateral or bilateral and semi-quantitatively graded by the number of X-ray zones (upper, mid and lower) presenting lesions. For unilateral lesions, “+” indicates involvement of 1 zone and “++” indicates involvement of more than 1 zone. For bilateral lesions, “+” indicates 2 zones, “++” indicates 3–4 zones, and “+++” indicates 5–6 zones. **(B–E)** Comparison of average values for first **(B, D)** and second **(C, E)** components in individual TB patients classified by presence or absence of cavities **(B, C)** and miliary TB versus other extrapulmonary TB (eTB) forms **(D, E)**. Statistical analysis was performed by two-tailed t-test for unpaired samples using Welch correction. Benjamini-Hochberg correction was applied to reduce the bias resulting from multiple testing.

We initially conducted a multivariate analysis integrating clinical, radiological, and laboratory indicators to define severity-related phenotypes in TB. To reduce dimensionality, and due to the mixed nature of the dataset (continuous and categorical variables), we applied Factor Analysis of Mixed Data (FAMD). This approach allowed us to identify the major axes of variation present in the dataset. The first two components captured the largest proportion of variance (22.41%) and revealed clinically interpretable patterns (**Figure 1A**).

In the lower-left quadrant of the plot, we found values typically associated with lower disease severity, such as normal serum albumin levels, small X-ray lesions limited to one lung (Unilateral lesions++) or even one lobe (Unilateral lesions+), and low sputum bacillary counts.

The upper-right quadrant was occupied by characteristic features of advanced post-primary disease, such as extensive lesions in both lungs (bilateral lesions+++), elevated levels of neutrophils and monocytes, purulent cough and high acid-fast bacilli (AFB) counts in sputum (AFB+++). On the other hand, the upper-left quadrant contained some values associated with moderate bacillary charge (AFB++) and small pulmonary lesions located in both lungs (bilateral lesions+), together with the absence of symptoms as night sweats, weight loss, fever and/or dyspnoea. These observations likely indicate a moderate severity and a possible transitional state to the more advanced disease characteristics located in the upper-right quadrant, which would be consistent with the asymptomatic alveolitis characteristic of early post-primary TB (12).

In the lower-right quadrant, features related to disseminated TB showed the largest contribution to both components. This included pleural effusion, extrapulmonary forms and symptoms such as weight loss, fever, night sweats and dyspnoea, together with absence of bacilli in sputum and dry cough, characteristic of severe primary TB.

Other variables such as AFB, type of cough and presence/absence of constitutional symptoms (malaise, fatigue, anorexia and intermittent low fever) were predominantly represented in the second component. Surprisingly, hemoptysis appeared in the quadrant we identified with mild disease (lower left). Further analyses, however, suggested that patients presenting this alarming symptom seek medical attention earlier in the course of the disease, presenting smaller pulmonary cavitary lesions and an overall milder condition (**Supplementary Figure 1**).

Taken together, the distribution of values in the plot suggests that the first component can be identified with a systemic type of severity, likely linked to bacterial dissemination whereas the second component reflects a severity more closely associated with “pulmonary” severity and bacillary burden (**Figure 1A**).

Studying the values of the first two components established by FAMD for individual patients, we observed a continuous distribution. However, individuals in the upper left quadrant clustered near zero, whereas those in the lower right quadrant showed the highest average values for the first component and lower values for the second. (**Supplementary Figure 2**).

To validate the FAMD analysis, we evaluated the association between the first two components and two variables not included in the construction of the multivariate analysis, in order to avoid circularity.

First, we compared TB patients with or without pulmonary cavities, a hallmark of post-primary disease associated with lung destruction and increased bacillary burden (45). We did not observe an association between the presence of cavities and the average scores of the first component (**Figure 1B**); however, cavitary patients showed higher values for the second component (**Figure 1C**).

We then examined patients with extra-pulmonary disease, comparing miliary TB with other, less severe extra-pulmonary forms. Miliary TB was associated with the highest scores on the first component (**Figure 1D**) and no differences were observed in the second (**Figure 1E**).

These results supported the clinical interpretability of the first two FAMD components and their association with disease severity.

Based on this analysis, we then defined an overall severity score for each individual as the weighted sum of the two components according to the percentage of variance explained.

### 3.3 Determination of plasma IgG levels against *Mtb* antigens in TB patients

**Figure 2:**
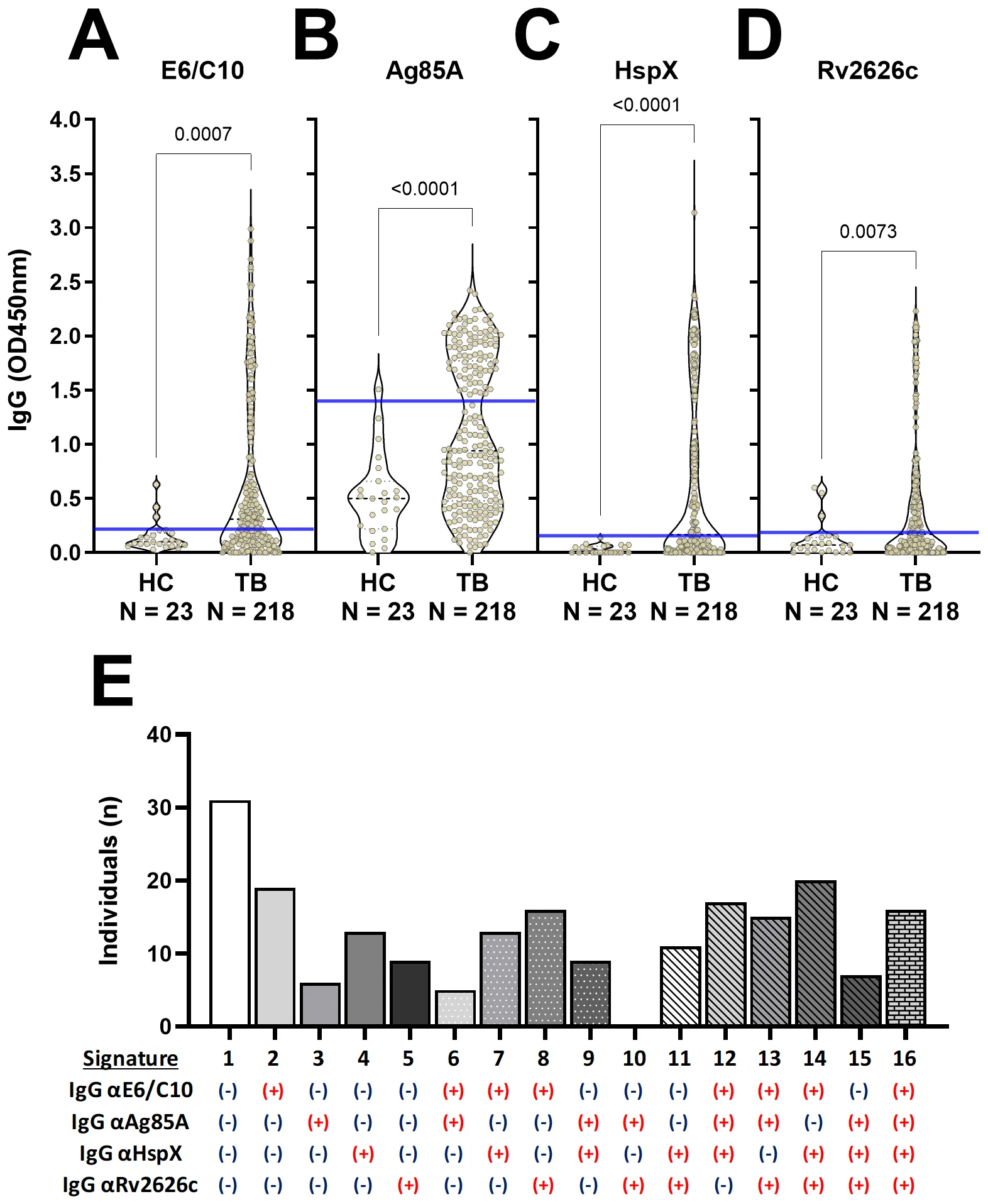
Levels of antibodies against *Mtb* antigens in TB and HC. **(A–D)** Levels of specific antibodies were determined in plasma by ELISA. Each dot represents the value of optical density at 450 nm obtained for an individual against E6/C10 **(A)**, Ag85A **(B)**, HspX **(C)** and Rv2626c **(D)**. Blue lines indicate the cut-off values used to define individuals with elevated antigen-specific IgG levels. Statistical analysis was performed by Mann-Whitney test. **(E)** Number of TB patients displaying the different antibody signatures analyzed (bars). Benjamini-Hochberg correction was applied to reduce the bias resulting from multiple testing.

We next measured plasma IgG antibodies against E6/C10, Ag85A, HspX, and Rv2626c in individuals with TB and in healthy controls (HC). Mean IgG levels were significantly higher in TB patients for all analyzed antigens (**Figures 2A–D**). It is important to note that anti-Ag85A antibody levels were higher than those against other antigens in both TB patients and HC, likely due to prior BCG vaccination, as immunity against Ag85A is induced by BCG vaccination but not against the other antigens (46). We then analyzed “antibody signatures”, defined as combinations of antigen-specific IgG responses. Surprisingly, we found that none of the TB patients analyzed (n = 218) exhibited IgG reactivity exclusively against Ag85A and Rv2626c (signature 10, **Figure 2E**). In contrast, all other possible antibody signature combinations were observed, suggesting that these antibodies may function as antagonistic indicators of infection status.

### 3.4 Association between IgG reactivity and disease severity

**Figure 3:**
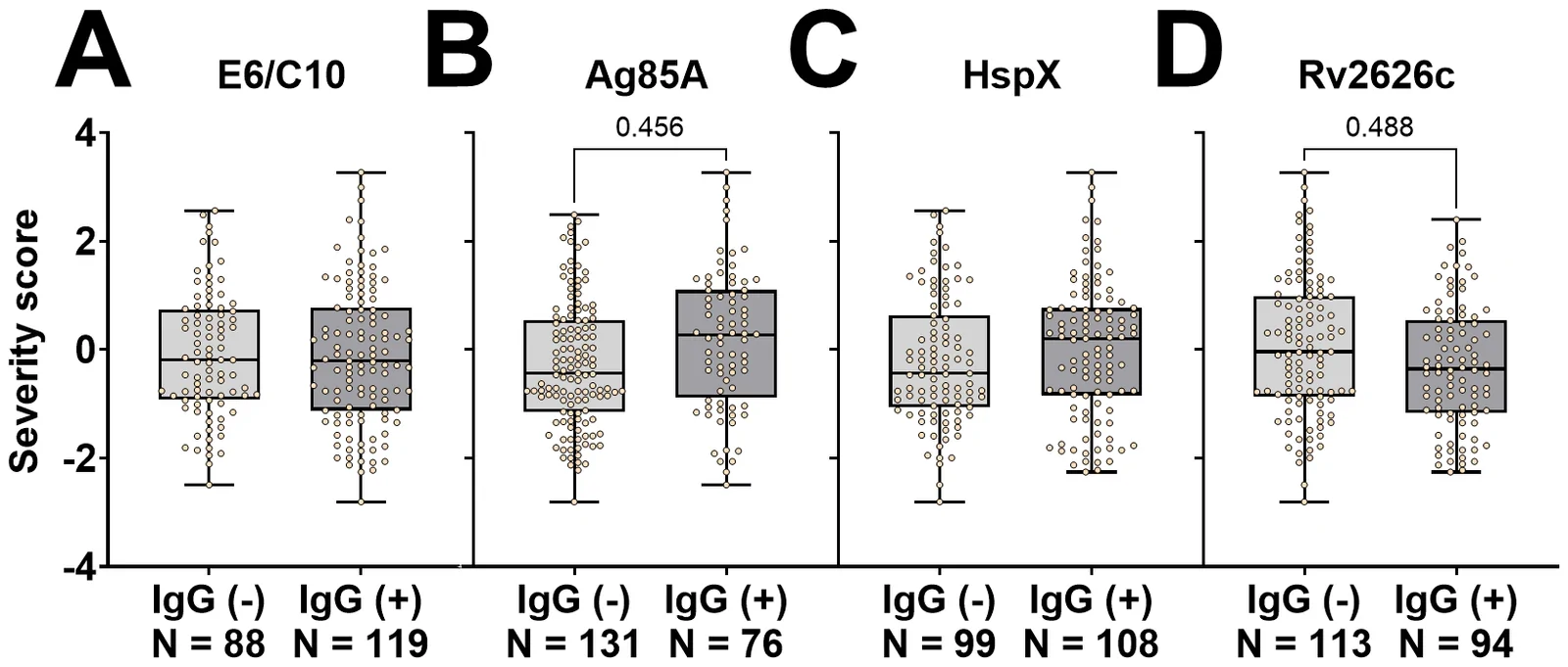
Severity score and IgG reactivity against individual antigens. **(A–D)** Severity score of TB patients classified by their presence (+) or absence (–) of increased IgG levels against E6/C10 **(A)**, Ag85A **(B)**, HspX **(C)** and Rv2626c **(D)**. Each dot represents the severity score of an individual TB patient, calculated as the weighted sum of the first and second components from the FAMD analysis, with weights corresponding to the percentage of variance explained by each component. Statistical analysis was performed by two-tailed t-test for unpaired samples, using Welch correction. Benjamini-Hochberg correction was applied to reduce the bias resulting from multiple testing.

Initially, a very slight increase in the average severity score was observed in TB patients with antibodies against Ag85A, whereas a slight decrease was detected in those recognizing Rv2626c (**Figure 3**). When severity-linked parameters were analyzed separately, no strong associations were detected for IgG against E6/C10, HspX and Rv2626c. However, IgG against Ag85A was associated with cavitary disease (**Supplementary Figure 3**). Considering this, we decided to study the relation between severity and the combination of antigens recognized.

**Figure 4:**
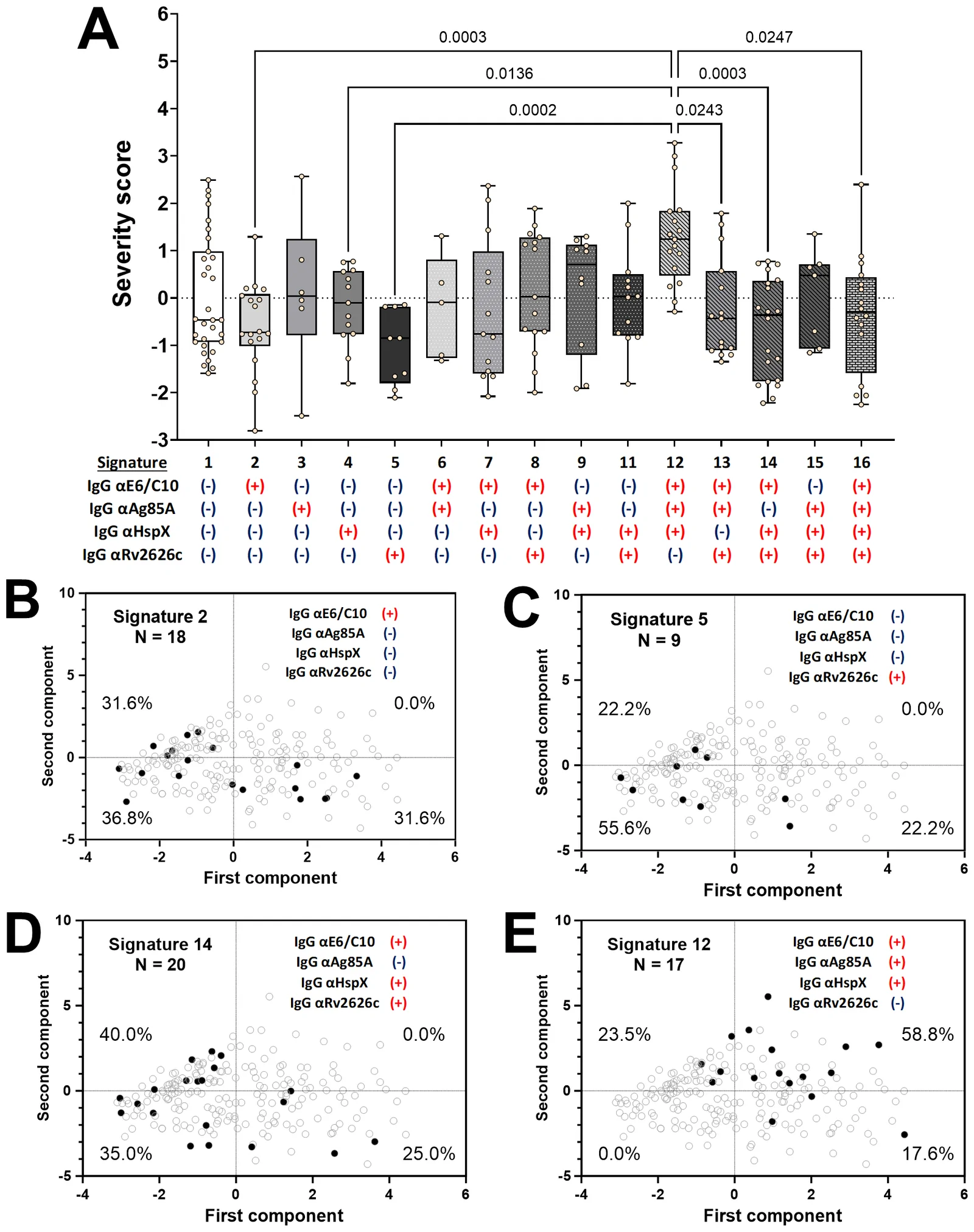
Antibody signatures and severity. **(A)** Severity score for individual TB patients classified by their antibody signatures, derived from presence (+) or absence (–) of IgG against each antigen. Each dot represents the value of severity score for an individual TB patient. Statistical analysis was performed by Brown-Forsythe and Welch ANOVA tests followed by Dunnett’s T3 multiple comparisons post-test. **(B–D)** Distribution of first and second component for individuals with signatures 2 **(B)**; 5 **(C)**; 14 **(D)** and 12 **(E)**. Each dot represents an individual. Black dots indicate TB patients presenting the corresponding signature and empty dots representing those with other signatures. Percentage of individuals within each quadrant are indicated for each signature, along with presence (+) or absence (–) of increased IgG levels against each antigen.

**Figure 4** shows the severity score of TB patients classified according to their antibody signatures. TB patients exhibiting increased IgG responses to Ag85A, E6/C10 and HspX but not to Rv2626c (Signature 12) presented the highest average severity scores. Most patients with responses limited to single antigens (Signatures 2, 4, 5) displayed significantly lower average scores, with the exception of Ag85A (Signature 3). The same was true for most individuals responding to three or more antigens (Signatures 13, 14, 16). These results suggest that specific antibody recognition patterns in TB have a complex but tangible relation with disease severity.

We next analyzed the antibody signatures associated with average scores significantly different from zero and examined their values in the first and second components of the FAMD. Signature 12 was selected for its high severity score (n = 17, Mean: 1.304, 95% CI: 0.777 to 1.832), whereas signatures 2 (n = 18, Mean: -0.538, 95% CI: -1.012 to -0.064), 5 (n = 9, Mean: -1.056, 95% CI: -1.662 to -0.4504) and 14 (n = 20, Mean: -0.639, 95% CI: -1.012 to -0.064) were selected among the lowest scores. Patients with signatures 2, 5 and 14 showed a similar distribution in the FAMD score plot. None of the individuals within these groups were located in the upper right quadrant (0%), associated with severe post-primary disease. In contrast, 33.3%, 55.6% and 35.0% of subjects with these signatures clustered in the lower left quadrant, corresponding to mild disease (**Figures 4B, C**). Conversely, more than 50% of the patients characterized by signature 12 were distributed in the upper right quarter while none were found in the lower-left quadrant (**Figure 4D**). These findings indicate that the increased average severity score associated with signature 12 was not due to contributions of either principal component in isolation.

**Figure 5:**
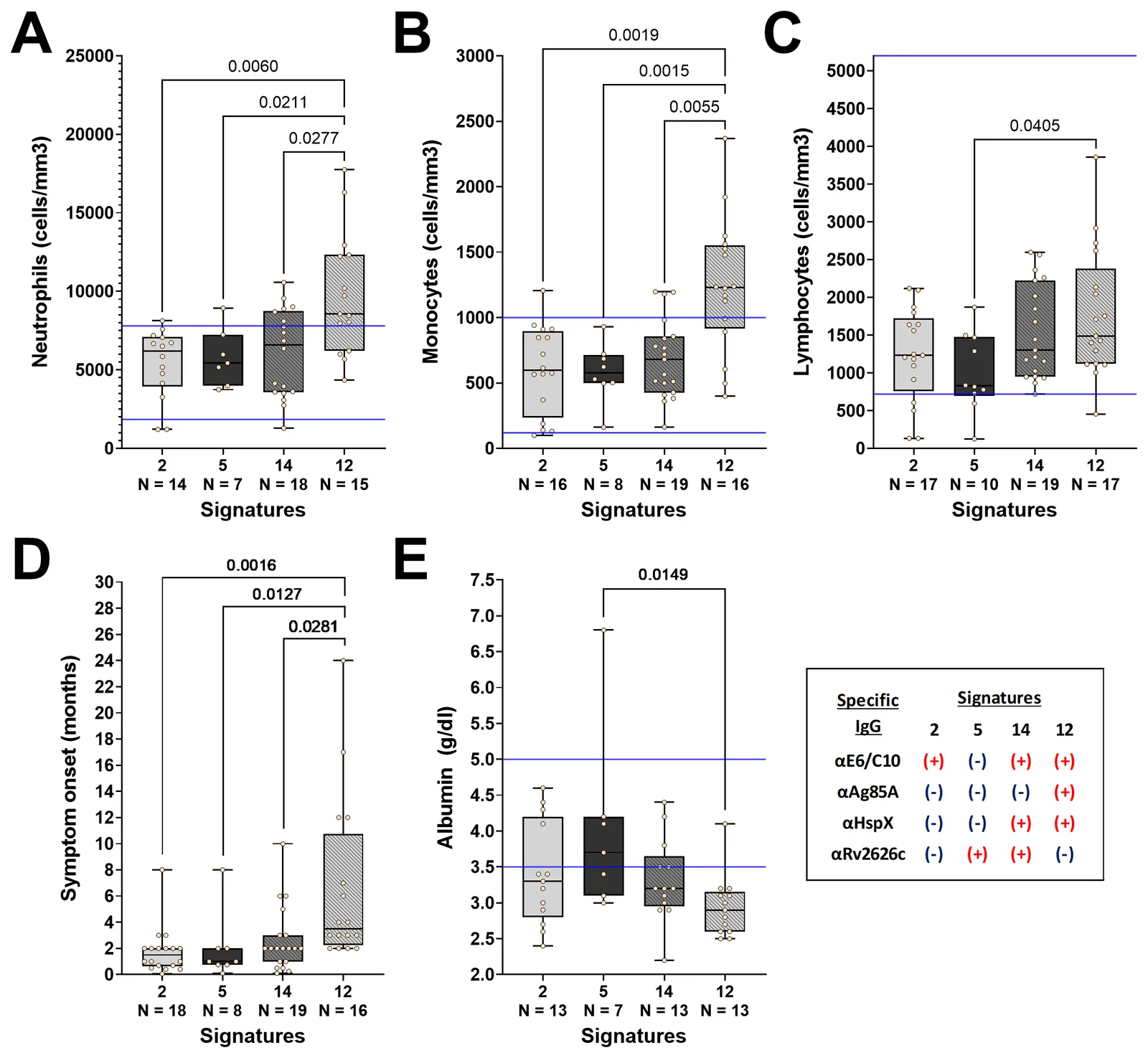
Characterization of selected antibody signatures through biochemical parameters. **(A)** Neutrophil counts. **(B)** Monocytes counts. **(C)** Lymphocyte counts. **(D)** Albumin levels in blood. **(E)** Estimated time since symptom onset for patients with the selected signatures. Statistical analysis was performed by Brown–Forsythe and Welch ANOVA were used, followed by Dunnett’s T3 post multiple comparisons test. Blue lines indicate reference ranges for healthy adults (47). Symptom onset and albumin levels were log-transformed prior to statistical analysis to satisfy the assumption of normality.

After identifying the four antibody signatures most strongly associated with overall severity through multivariate analysis, we examined individual parameters (included or not in the model) to further characterize them. Patients displaying signature 12 were significantly more likely to exhibit bilateral lesions on chest radiography and to present productive cough, being the only ones displaying purulent cough. On the other hand, these patients were less likely to present hemoptysis than those with signatures 5 or 14 (**Table 2**). Patients with signature 12 also showed a significantly longer time since symptom onset than the three milder signatures (2, 5 and 14). Moreover, they presented elevated counts of neutrophils and monocytes, while most individuals with signatures 2, 5 and 14 did not exceed normal reference ranges (47). Patients with signature 5 in particular presented higher levels of albumin and lower levels of lymphocytes compared to those with signature 12 (**Figure 5**). Patients with signature 14 on their part, presented a smaller percentage of individuals with high AFB counts in sputum, compared to those with signature 12, with a similar, non-significant tendency being observed in signatures 2 and 5 (**Table 2**). Taken together, our results indicate that signature 12 is associated with advanced, post-primary disease, whereas signatures 2, 5 and 14 are linked to less advanced phenotypes

**Table 2:** Characterization of selected antibody signatures through radiological and clinical features.

Parameters		Signature 2		Signature 5		Signature 14		Signature 12
		n (%)	*P* -value	n (%)	*P* -value	n (%)	*P* -value	n (%)
Cavitation	(–)	6 (33.3%)	0.343	4 (40%)	0.441	6 (27.3%)	0.258	2 (11.1%)
	(+)	12 (66.7%)		6 (60%)		16 (72.7%)		16 (88.9%)
Pleural effusion	(–)	17 (89.5%)	>0.999	8 (80%)	0.852	18 (90%)	>0.999	17 (94.4%)
	(+)	2 (10.5%)		2 (20%)		2 (10%)		1 (5.6%)
Bilateral lesions	(–)	12 (66.7%)	**<0.001**	6 (66.7%)	**0.002**	12 (54.5%)	**0.003**	1 (5.9%)
	(+)	6 (33.3%)		3 (33.3%)		10 (45.5%)		16 (94.1%)
Constitutional symptoms	(–)	8 (42.1%)	0.453	5 (55.6%)	0.581	7 (36.8%)	0.481	4 (23.5%)
	(+)	11 (57.9%)		4 (44.4%)		12 (63.2%)		13 (76.5%)
Hemoptysis	(-)	12 (66.7%)	0.088	3 (33.3%)	**0.007**	12 (57.1%)	**0.019**	16 (94.1%)
	(+)	6 (33.3%)		6 (66.7%)		9 (42.9%)		1 (5.9%)
Weight loss	(-)	14 (77.8%)	0.123	6 (66.7%)	0.411	16 (76.2%)	0.069	7 (41.2%)
	(+)	4 (22.2%)		3 (33.3%)		5 (23.8%)		10 (58.8%)
Cough of any type	(-)	12 (66.7%)	0.066	7 (77.8%)	0.113	12 (57.1%)	0.111	5 (29.4%)
	(+)	6 (33.3%)		2 (22.2%)		9 (42.9%)		12 (70.6%)
Cough with sputum	(-)	13 (92.9%)	**0.002**	7 (87.5%)	**0.041**	13 (72.2%)	**0.037**	5 (31.3%)
	(+)	1 (7.1%)		1 (12.5%)		5 (27.8%)		11 (68.8%)
Purulent cough	(-)	14 (100%)	0.067	8 (100%)	0.130	18 (100%)	**0.047**	11 (68.8%)
	(+)	0 (0%)		0 (0%)		0 (0%)		5 (31.3%)
Dyspnoea	(-)	14 (77.8%)	>0.999	8 (88.9%)	>0.999	17 (81%)	>0.999	13 (76.5%)
	(+)	4 (22.2%)		1 (11.1%)		4 (19%)		4 (23.5%)
Fever	(-)	14 (77.8%)	>0.999	6 (66.7%)	>0.999	20 (95.2%)	0.214	12 (70.6%)
	(+)	4 (22.2%)		3 (33.3%)		1 (4.8%)		5 (29.4%)
Night sweats	(-)	12 (66.7%)	0.472	6 (66.7%)	0.429	16 (76.2%)	0.283	8 (47.1%)
	(+)	6 (33.3%)		3 (33.3%)		5 (23.8%)		9 (52.9%)
High AFB	(-)	12 (63.2%)	0.181	8 (80%)	0.077	18 (85.7%)	**0.014**	6 (37.5%)
	(+)	7 (36.8%)		2 (20%)		3 (14.3%)		10 (62.5%)

Individuals from each signature presenting “(+)” or not “ (–)” each feature are represented as “number (percentage)”. Statistical analysis was performed by Fisher exact test comparing individuals with signature 2, 5 or 14 against those with signature 12. Benjamini-Hochberg correction was applied to reduce the bias resulting from multiple testing.*P*-values <0.05 are written in bold.

## 4 Discussion

TB is a complex disease with heterogeneous clinical presentations and a wide spectrum of progression. Patients may present focal infiltrates, nodular or consolidative lesions, cavitary disease and a wide variety of disseminated forms; as a consequence of diverse host-pathogen interactions. Accordingly, previous reports have employed multivariate analysis to characterize immunological phenotypes and link them to clinical parameters (19). However, to our knowledge, no previous study has integrated clinical, radiological, and laboratory variables to characterize disease severity phenotypes by using FAMD analysis in TB.

It is known that hypoalbuminemia is a marker of TB severity, in association with increased mortality (48), treatment failure (49) and high lung bacillary burden (45). Moreover, high neutrophil and monocyte counts together with low lymphocyte numbers are predictors of residual pulmonary damage after treatment (50, 51), also associated with hypoalbuminemia (52). Furthermore, Panteleev et al. demonstrated that elevated circulating neutrophils and diminished lymphocyte levels are directly linked to bilateral pulmonary lesions, pulmonary destruction and high AFB counts (53), whereas Luo et al. found that monocyte counts correlate with the size of pulmonary cavities (50). Our interpretation of FAMD first and second components as related to systemic and pulmonary severity is consistent with these previous reports. In particular, albumin contributes negatively to both components; whereas monocytes and neutrophils counts are positively associated with the second component.

On the other hand, extrapulmonary TB has been associated with increased hospitalization times, marked weight loss and decreased sputum production (52). In addition, pleural effusion is linked to fever, dyspnea, dry cough and low bacillary burden (14). These features are also consistent with our results, contributing positively to the FAMD first component (systemic severity) and negatively to the second (pulmonary severity).

Therefore, having established a clinically interpretable framework for characterizing TB severity, we next examined whether antigen-specific antibody responses could provide additional information about disease phenotypes. The antigens studied in this work present distinct patterns of expression/secretion in relation to the metabolic state of *Mtb*, suggesting that antibody signatures may be influenced by host immune responses linked to disease severity. Previous reports have found increased levels of IgG against E6/C10 (22, 26, 27), Ag85A (20, 24, 25), HspX (22, 23, 26, 27) and Rv2626c (20–22) in some TB patients. However, antibodies from TB patients have been reported to lack the protective efficacy displayed by some exposed asymptomatic individuals, suggesting that they may function as indicators of disease states rather than actually causing them (54). Indeed, antibody responses against individual antigens have been shown to be informative or even show prognostic value. In this line, Baumann et al. reported that elevated anti-ESAT-6 antibody levels were associated with a slower response to TB treatment (55). Furthermore, Mattos et al. found that these antibody levels decreased during treatment, whereas IgG responses against HspX increased transiently before subsequently declining (27).

Considering previous reports and taking into account our results, we hypothesize that IgG responses against each of the four studied antigens would provide different types of information about TB infection. In this way, E6/C10 would function as an indicator of bacillary load and overall metabolic activity, being induced in fast growing (moderate or mild) or late (severe) disease, due to its constitutive expression (29). Responses against Ag85A would be associated with the presence of aerobic, replicating bacilli (29); which would explain their association with cavitation and increased severity, particularly as this antigen is highly induced when bacilli in NRP reactivate after exposure to increased oxygen (9). Lastly, responses against both HspX and Rv2626c would indicate the presence of bacilli in NRP but their respective functions may suggest an explanation for the difference observed in their behavior. HspX, has been found to be located extracellularly during early infection (56) and disease (40) but shift to the intracellular space when hypoxic environments are established (57), consistent with its dual functions in immune suppression (41) and persistence (39). In contrast, Rv2626c effects over macrophage phenotypes (7) and necrosis (6) suggest a possible role for its secretion in persistence and reactivation. These features indirectly associate responses against Rv2626c with immune control. Moreover, Rv2626c would present an opposite pattern of secretion to that of Ag85A, being directly induced by immune control (3, 38) and inhibited by reactivation (9). This further supports the idea of a limited antagonism between these antigens and suggests that IgG against Rv2626c may indicate a certain capacity from the immune system to restrict TB pathology, perhaps by limiting the growth of existing lesions or suppressing new foci of infection, which would explain its association to lower severity scores.

Among the antibody signatures analyzed, signature 12 was strongly associated with increased disease severity in the context of a severe post-primary phenotype. This signature is characterized by increased IgG levels against E6/C10, Ag85A and HspX but not Rv2626c. These observations suggest that signature 12 may represent a disease state characterized by extensive pulmonary pathology and heterogeneous bacterial metabolic activity. On the opposite end, signatures characterized by isolated responses against E6/C10, HspX or Rv2626c would indicate different forms of early TB, in line with our observations for symptom onset and severity. We didn’t manage to identify a signature related to severe primary TB. As mentioned, both Rv2626c and HspX antigens are induced during NRP and the isolated responses to HspX (signature 4) or Rv2626c (signature 5) were associated with lower disease severity. However, only Rv2626c maintained this association when combined with Ag85A and E6/C10. These observations could be explained by a differential post-transcriptional regulation of these antigens, consistent with the available evidence (6, 7, 40, 56, 57).

Nevertheless, a limitation of our study is the lack of follow-up of patients, which would allow us to evaluate the temporal evolution of antibody signatures and their possible association with treatment outcomes. Indeed, longitudinal studies will be required to determine whether these antibody signatures may have prognostic value as surrogate markers, for example, to predict treatment response, disease progression, or residual lung damage.

Overall, our results demonstrate that antigen-specific antibody signatures provide clinically significant information about tuberculosis disease states. Taking into account immune responses with clinical and radiological parameters, our study suggests that antibody signatures could show aspects of the metabolic heterogeneity of *Mtb* active infection. The identification of antibody signatures associated with different severity phenotypes raises the possibility for future studies to evaluate serological profiles as biomarkers to stratify tuberculosis patients, studying both their evolution and relation to outcomes. Thus, antibody signatures could contribute to a better phenotypic characterization of TB patients and, potentially, to the development of better diagnostic and prognostic strategies.

## Data Availability

The raw data supporting the conclusions of this article will be made available by the authors, without undue reservation.
