# Supplementary material for: Circulating antibody signatures against Mycobacterium tuberculosis antigens are associated with severe and mild tuberculosis
Source: Front Immunol. 2026 Aug 25;17:1897502. doi: 10.3389/fimmu.2026.1897502 (PMC13549925; doi:10.3389/fimmu.2026.1897502)
Supplement: Supplementary file 1 [file DataSheet1.pdf]

Circulating antibody signatures against *Mycobacterium tuberculosis* antigens are associated with severe and mild tuberculosis
(Supplementary Material)

Supplementary Figure 1. Hemoptysis is associated with milder disease. TB patients presenting or not hemoptysis were compared with regards to months since symptom onset (A), age (B), presence and distribution of cavities in lung X-rays (C), AFB in sputum greater or lesser than 10 bacilli per field (D) and levels of neutrophils (E), monocytes (F), lymphocytes (g albumin (H) and hemoglobin (I) in blood. Statistical analysis was performed by two-tailed t-test for unpaired samples, using Welch correction in the case of numerical variables (A, B, E, F, G, H, I) and chi-square test in the case of categorical variables (C, D).

Supplementary Figure 2. Distribution of individuals in the FAMD plot. (A) Distribution of values for first and second component from the FAMD analysis in our population of TB patients. Each individual is represented as a dot. Upper left (UL), upper right (UR), lower left (LL) and lower right (LR) quadrants are indicated. (b-c) Average values of first (B) and second (C) components for individuals from the different quadrants of the FAMD plot. Statistical analysis was performed by Brown-Forsythe and Welch ANOVA tests followed by Dunnett's T3 multiple comparisons post-test, comparing Left and right quadrants separately for the first component (B) and Lower and upper quadrants for second component (C).

Supplementary Figure 3. Severity and specific IgG. Average symptom onset for TB patients classified by whether they present (+) or not (-) increased levels of IgG against E6/C10 (A), Ag85A (B), HspX (C) and Rv2626c (D). Levels of specific IgG against E6/C10 (E), Ag85A (F), HspX (G) and Rv2626c (H) in TB patients with or without cavities in their lungs. Each dot represents an individual. Statistical analysis was performed by two-tailed t-test for unpaired samples using Welch correction. Benjamini-Hochberg correction was applied to reduce the bias resulting from multiple testing.
